# Network Topology Analysis of Post-Mortem Brain Microarrays Identifies More Alzheimer’s Related Genes and MicroRNAs and Points to Novel Routes for Fighting with the Disease

**DOI:** 10.1371/journal.pone.0144052

**Published:** 2016-01-19

**Authors:** Sreedevi Chandrasekaran, Danail Bonchev

**Affiliations:** Center for the Study of Biological Complexity, Virginia Commonwealth University, Richmond, Virginia, United States of America; Texas Tech University Health Science Centers, UNITED STATES

## Abstract

Network-based approaches are powerful and beneficial tools to study complex systems in their entirety, elucidating the essential factors that turn the multitude of individual elements into a functional system. In this study we used critical network topology descriptors and guilt-by-association rule to explore and understand the significant molecular players, drug targets and underlying biological mechanisms of Alzheimer’s disease. Analyzing two post-mortem brain gene microarrays (GSE4757 and GSE28146) with Pathway Studio software package we constructed and analyzed a set of protein-protein interaction, as well as miRNA-target networks. In a 4-step procedure the expression datasets were normalized using Robust Multi-array Average approach, while the modulation of gene expression by the disease was statistically evaluated by the empirical Bayes method from the limma Bioconductor package. Representative set of 214 seed-genes (p<0.01) common for the three brain sections of the two microarrays was thus created. The Pathway Studio analysis of the networks built identified 15 new potential AD-related genes and 17 novel AD-involved microRNAs. Using KEGG pathways relevant in Alzheimer’s disease we built an integrated mechanistic network from the interactions between the overlapping genes in these pathways. Routes of possible disease initiation process were thus revealed through the CD4, DCN, and IL8 extracellular ligands. DAVID and IPA enrichment analysis uncovered a number of deregulated biological processes and pathways including neuron projection/differentiation, aging, oxidative stress, chemokine/ neurotrophin signaling, long-term potentiation and others. The findings in this study offer information of interest for subsequent experimental studies.

## Introduction

In 1906, Dr. Alois Alzheimer was the first one to identify and report the link between worsening psychological symptoms and the histopathological modifications in the brain [1]. By late 1970s, Alzheimer’s disease (AD) was recognized as the most common form of dementia (serious progressive loss of cognitive functions), as well as one of the leading fatal conditions affecting elderly population. Initially AD patients start out with memory loss which then manifests into confusion and disorientation followed by slow and progressive decline in their standard of daily living. The disease is characterized by the presence of amyloid-plaques (which consist of amyloid-β peptides) and neurofibrillary tangles (NFTs) in the brain. In general, AD etiology is sporadic but a small percentage of early-onset familial form is also possible. Mutations in APP, APOE, PSEN1, PSEN2 and MAPT genes were found to cause Alzheimer’s disease pathogenesis.

We present in S1 Fig which depicts the various genes already implicated in Alzheimer’s disease along with different deregulated biological processes caused by the several abnormal protein activities. Many biological processes including neuroinflammation, oxidative stress, dysfunction of lysosomal/proteasomal degradation, mitochondrial dysfunction etc., have been associated with Alzheimer’s disease [2–11]. Aggregation of amyloid-plaques and tau proteins were suggested to be the major cause for these deregulations [11–14]. Among many dysfunctional genes the following genes have been found play critical role in the Alzheimer’s disease manifestation. APP encodes for the amyloid-β precursor protein, and the presenilin genes (PSEN1 and PSEN2) encode for the proteolytic enzymes PS1 and PS2 that cleave APP into amyloid-β and other fragments [15]. PSEN1 mutations were attributed to more than 50% of early onset of familial form of AD [16]. Neurofibrillary tangles consist of aggregations of hyperphosphorylated tau proteins (MAPT) [13,17]. Apart from these genes, APOE, CDK5, LMO4, PTEN, TGFβ 1 etc., were found to increase the abnormal protein aggregation and other characteristic features of Alzheimer’s disease (Brains of individuals with AD manifest two characteristic lesions: extracellular amyloid plaques and intracellular neurofibrillary tangles of hyperphosphorylated tau protein. Amyloid plaques contain small, toxic cleavage products (denoted as amyloid β40 and amyloid β)) [18–22].

In addition, the role of miRNA regulatory mechanisms was also studied in Alzheimer’s disease. A recent genome-wide miRNA profiling study has found a substantial number of differentially expressed miRNAs in the cortical region of AD brains. Many of these miRNAs and their predicted mRNA target pairs were part of several biological processes that were previously reported dysfunctional in Alzheimer’s disease mechanism [23].

Owing to several sophisticated biomolecular research techniques many more deregulated genes and biological processes have been identified and reported in this disease paradigm. Our study is a modest effort to understand and explore the underlying cellular mechanisms and critical molecular players, as well as to identify novel drug targets using comprehensive network-based analysis. In this way, one could study these kinds of complex disease models with all its individual players along with the interactions among them. Network-based analysis is a valuable tool to understand and appreciate the underlying complexity of a system as a whole rather than disconnected unit. This systems biology approach allows searching for unifying patterns and possibly common mechanisms of complex diseases frequently involving distinct and heterogeneous datasets. The present research work on Alzheimer’s disease is part of a broader network-based data analysis of three neurodegenerative disorders (NDDs) including Parkinson’s (PD) and Huntington’s disease (HD) with the ultimate goal of identifying in a future study a unified underlying molecular mechanism of these three devastating NDDs. The manuscript outlining our network-based analysis findings of Parkinson’s disease was published earlier [24]. Few recent research works have utilized similar network-based approach to identify candidate genes for Alzheimer’s disease [25–27].

## Materials and Methods

The network-based analysis of the three neurodegenerative diseases under study was performed by following the same rationale and work flow. For more specific details, refer the *Methods and data* part of our Parkinson’s disease network analysis paper [24]. This section will briefly explain the different steps involved in the study data collection and analysis.

The network-based analysis of Alzheimer’s disease comprises of six steps, beginning with the identification of microarray gene expression datasets. For this, we searched both National Center of Biotechnology Information’s (NCBI) Gene Expression Omnibus (GEO) and European Bioinformatics Institute’s (EBI) ArrayExpress databases for Affymetrix microarray gene expression datasets of Alzheimer’s disease. Two datasets namely GSE4757 [28] and GSE28146 [29] which contains post-mortem brain tissue samples from diseased (patients with AD) and control (normal) conditions were found and used for this research work. The first one is related to entorhinal cortex and the second one uses CA1 region in hippocampus. These two regions in the brain play important role in memory formation, which is essential in diagnostics since memory loss and disorientation are the early signs of AD. The GSE4757 dataset had expression profile of both neurons containing neurofibrillary tangles and normal neurons from the entorhinal cortex of 10 mid-stage AD cases. The GSE28146 had 30 samples of different stages of AD cases including controls. For detailed sample information and other data, refer the NCBI GEO website and the contributor’s journal articles [28–31].

Secondly, the selected microarray expression datasets were normalized using Robust Multi-array Average (RMA) approach [32]. The differential gene expression changes were statistically evaluated by the empirical Bayes (eBayes) method [33] from the limma Bioconductor package. Probe-sets with p-values < 0.05 were considered to be significantly differentially expressed genes (SDEGs). The statistical analysis was carried out using in-house written R software programming code utilizing Bioconductor package [34]. The R program source code is available upon request by the author (SC).

**Fig 1:**
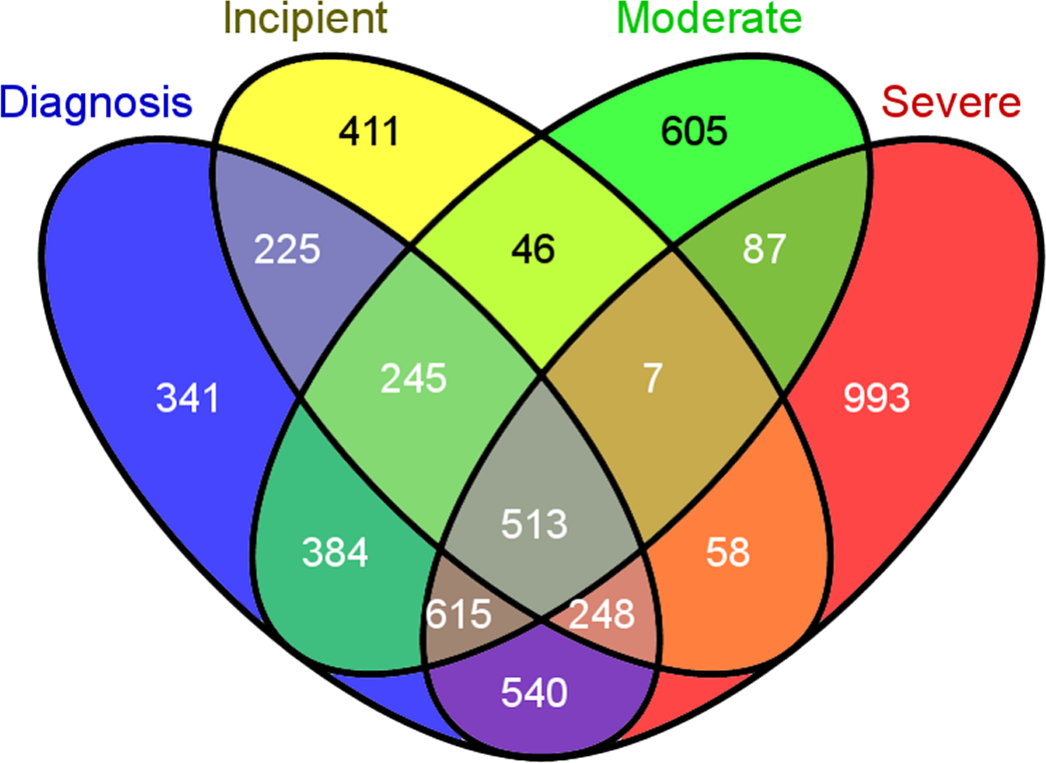
Four-set Venn diagram of the overlap of significantly differentially expressed genes (SDEGs) in GSE28146 gene expression datasets. Courtesy: Oliveros, J.C. (2007–2015) Venny. An interactive tool for comparing lists with Venn's diagrams. Publicly available at http://bioinfogp.cnb.csic.es/tools/venny/index.html.

Then the two microarray datasets were subjected to the same statistical analysis and the significantly differentially expressed gene lists called “seed genes” were generated for each dataset. The statistical analysis of GSE4757 dataset resulted in finding 725 (p-values < 0.05) SDEGs between AD cases and control samples. The lists generated from the GSE28146 dataset were denoted as incipient, moderate and severe, for the three stages of Alzheimer’s disease condition. In addition, differential gene expression changes found between control and AD cases irrespective of disease stage were denoted as “Diagnosis”. An overlap of 513 (p-values < 0.05) seed genes was found between the four sets of SDEGs as shown in Fig 1. By combining the two seed genes lists and removing duplicates, a total of 1205 genes were found to be significantly differentially expressed between control and AD cases. In order to have a considerable number of overlapping genes as well as to partially compensate for not accounting for the multiple correlation, only those SDEGs with the p-value lower than 0.01 were included in network evaluation. Following this cut-off criteria, 214 genes were treated as “seed genes” which were then subjected to comprehensive network analysis. (See S1 Table for the list of 214 SDEGs).

The fourth step involves the construction of various networks such as *direct interaction*, *shortest-path* and *miRNA* regulation using Pathway Studio 9.0 software package [35], (http://www.elsevier.com/online-tools/pathway-studio). This software includes a proprietary molecular interaction database namely ResNet 9.0 (released October 15, 2011) that has curated data of various molecular interactions like binding, chemical reaction, direct regulation, expression, miRNA regulation, molecular synthesis, molecular transport, promoter binding, protein modification and regulations, as well as information about thousands of custom built cell-processes, metabolic and signaling pathways. Apart from these networks, key topological characteristics such as node degree (local connectivity), closeness centrality (network monitoring) and betweenness centrality (traffic-influential) scores were also calculated using the Pajek software package [36,37]. Based on these beneficial topological characteristics, along with their biological/ molecular functions relevant for the neurodegenerative process, the genes have been categorized as “already known AD-genes” and “genes of interest for AD”. The distinction was made by using sources like Online Mendelian Inheritance in Man (OMIM) database (http://omim.org/), NCBI’s PubMed database (http://www.ncbi.nlm.nih.gov/pubmed.com), MalaCards database (http://malacards.org/), and Google search for the latest publications (http://www.google.com). Each of these two categories were further divided in two subcategories, those found among the significantly differentially expression genes (SDEGs) and such emerging from the connecting proteins in *shortest-path* network.

Next step was to subject the seed genes to Gene Ontology (GO) enrichment analysis as it provides biological functional interpretation of large lists of genes derived from genomic studies such as microarray, proteomics experiments. This analysis was accomplished using Database for Annotation, Visualization and Integrated Discovery (DAVID), a widely used web-based application focusing on GO classification [38–40]. Along with the biological functional interpretation, interesting information was gathered about the different biological pathways that could be affected in Alzheimer’s disease. For this, we used the core analysis in Ingenuity’s IPA (Ingenuity Systems, http://www.ingenuity.com/) and Pathway Enrichment Analysis in Pathway Studio. Both the tools provided valuable information on several canonical pathways deregulated in Alzheimer’s disease.

Finally, an *integrated* mechanistic disease network was constructed using the genes/proteins found in common in all enriched Kyoto Encyclopedia of Genes and Genomes (KEGG) pathways resulted from DAVID analysis [41]. Again, Pathway Studio software was used to construct the *direct interaction* network using the “mechanism genes” (genes found in common in all enriched KEGG pathways) to investigate the *integrated* Alzheimer’s disease mechanism.

## Results

The following network-based analysis was implemented using the 214 “seed genes” resulted from the statistical analysis of the two microarray gene expression datasets (GSE4757 and GSE28146). To the best of our knowledge this is the first study to carry out such an analysis of Alzheimer’s disease utilizing the above mentioned datasets. We constructed various types of network to identify critical molecular players and mechanisms involved in AD.

### Alzheimer’s disease *direct* interaction network

**Fig 2:**
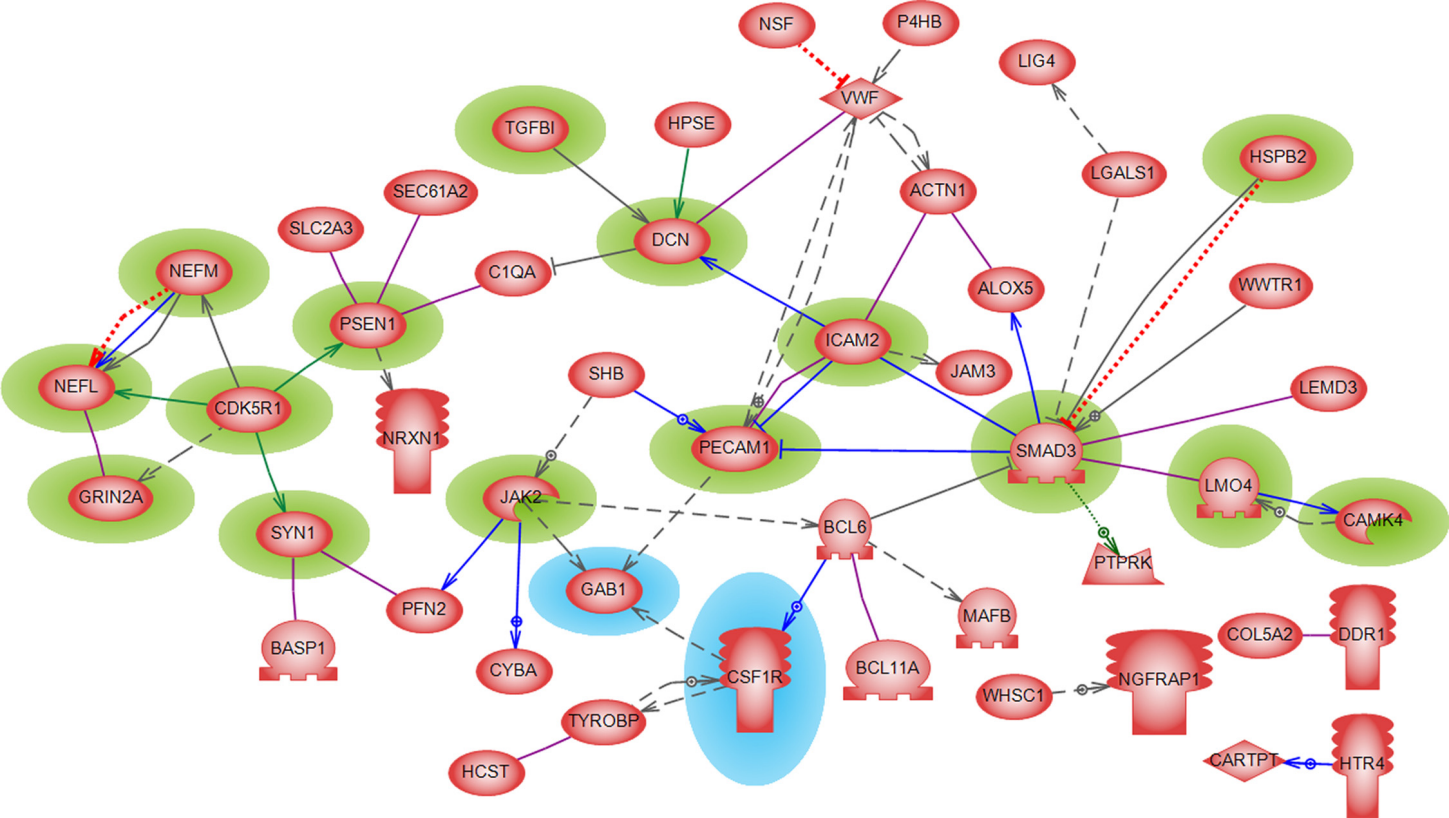
Alzheimer’s disease *direct interaction* network. The 15 genes/proteins implicated in AD pathology are highlighted in green and the two genes/proteins of potential interest for that disease are highlighted in blue.

As the name suggests, this network describes the *direct interaction* between any two genes/proteins, if any. Out of the 214 significantly differentially expressed genes (SDEGs), 48 genes were directly connected to each other based on the different interaction types like regulations, promoter binding, direct regulation, protein modification and microRNA’s regulation. This interaction network (Fig 2) has an average node degree of 2.20 with BCL6, DCN, JAK2, PECAM1 and SMAD3 as top five hub genes. Some of these hub genes (BCL6, DCN and SMAD3) along with C1QA and PSEN1 (a well-known gene to cause familial early-onset of AD) have high betweenness centrality score which position them among the top influential nodes in the network. 15 out of 48 SDEGs (CAMK4, CDK5R1, DCN, GRIN2A, HSPB2, ICAM2, JAK2, LMO4, NEFL, NEFM, PECAM1, PSEN1, SMAD3, SYN1 and TGFBI) are already associated in Alzheimer’s disease pathology. Additionally, two genes namely CAMK4, CSF1R and GAB1 from our SDEGs set could be considered as potentially related to AD mechanism based on their biological function.

#### Molecular functions of some well-known and candidate genes

The molecular functions of some genes that were known to contribute the neurodegeneration process in Alzheimer’s disease were briefly explained below. CDK5R1 is an activator of CDK5, a known gene that leads to the accumulation of aberrantly phosphorylated protein tau, a classic feature of AD pathology. From the *direct interaction* network (see Fig 2), GRIN2A, NEFM, NEFL and SYN1 genes were first level interacting partners of CDK5R1. Their regional alteration and reduced expression were thought to influence the severity of Alzheimer’s disease [42–44]. The network also revealed the direct interaction of CDK5R1 with PSEN1 (well-known as important AD regulator), as provided by the Pathway Studio ResNet database: Cyclin-dependent kinase-5/p35 phosphorylates Presenilin 1 to regulate carboxy-terminal fragment stability. CAMK4 is regulated by the Wnt signaling pathway and its expression could play a role in the neuroprotective function of the Wnt signaling against the Alzheimer's amyloid peptide. This role was confirmed in mouse AD model studies [45,46].

With their innate physiological roles as well as being *direct interacting* partners (guilt-by-association) to already known AD genes (JAK2 and PECAM1) increases the chances of the two genes (CSF1R and GAB1) as novel candidate genes for Alzheimer’s disease. Many suggest that oxidative stress is involved in the pathogenesis of most of the neurodegenerative disorders, including AD [12,48–52]. GAB1 has been identified as an important component in oxidative stress signaling and influencing the cell survival [47]. Even though CSFIR (colony stimulating factor 1 receptor) is a second-level interacting partner to AD known gene, based on its intrinsic neuroprotective function, it could also be a potential gene of interest in Alzheimer’s disease condition. Recent study show that CSF1R depleted mice exhibit increased neuronal loss after injury and vice versa. Its ligands CSF1 and IL34 exhibit neuroprotection against excitotoxicity induced by neurodegeneration [53]. Thus suggesting, up-regulation of CSF1R could play potential role in neuronal survival after injury and degeneration.

### Alzheimer’s disease *shortest-path* network (SPNW)

Unlike *direct interaction* network, *shortest-path* network (SPNW) enables us to find indirect relationship between two or more entities through intermediary nodes in the absence of direct relationship. One could identify genes/proteins that might contribute to the neurodegenerative process based on the inference (guilt-by-association) that when genes/proteins with well-defined biological functions (e.g., oxidative stress) interact with other genes/proteins, the latter have a higher probability to share that function, as compared to those selected at random [54,55].

One limitation of the *shortest-path* network is that sometimes it could bring in more intermediary nodes in order to have a unified network. Therefore to have a concise and yet meaningful network, we selected only those SDEGs with node degree ≥ 25 as given in the ResNet database of the Pathway Studio software. This cut-off criterion was satisfied by 96 out of 214 SDEGs and the resulted *shortest-path* network included an additional of 131 software-added from the ResNet database connecting genes/proteins. Following our categorization technique (See Methods and Data section), the 96 SDEGs along with the 131 software-added connecting genes were classified into the genes that were already related to AD and the genes that could be of potential interest in AD diagnosis and medical treatment. Table 1 summarizes the different categories and the corresponding numbers of genes.

**Table 1:** Summary of genes of interest and genes already known in Alzheimer's disease.

Different categories	No. of genes	Node color code in figures
Genes of interest from SDEGs	4	Blue
Known AD genes from SDEGs	18	Green
Genes of interest in SPNW connecting nodes	23	Orange
Known AD genes in SPNW connecting nodes	27	Red

**Fig 3:**
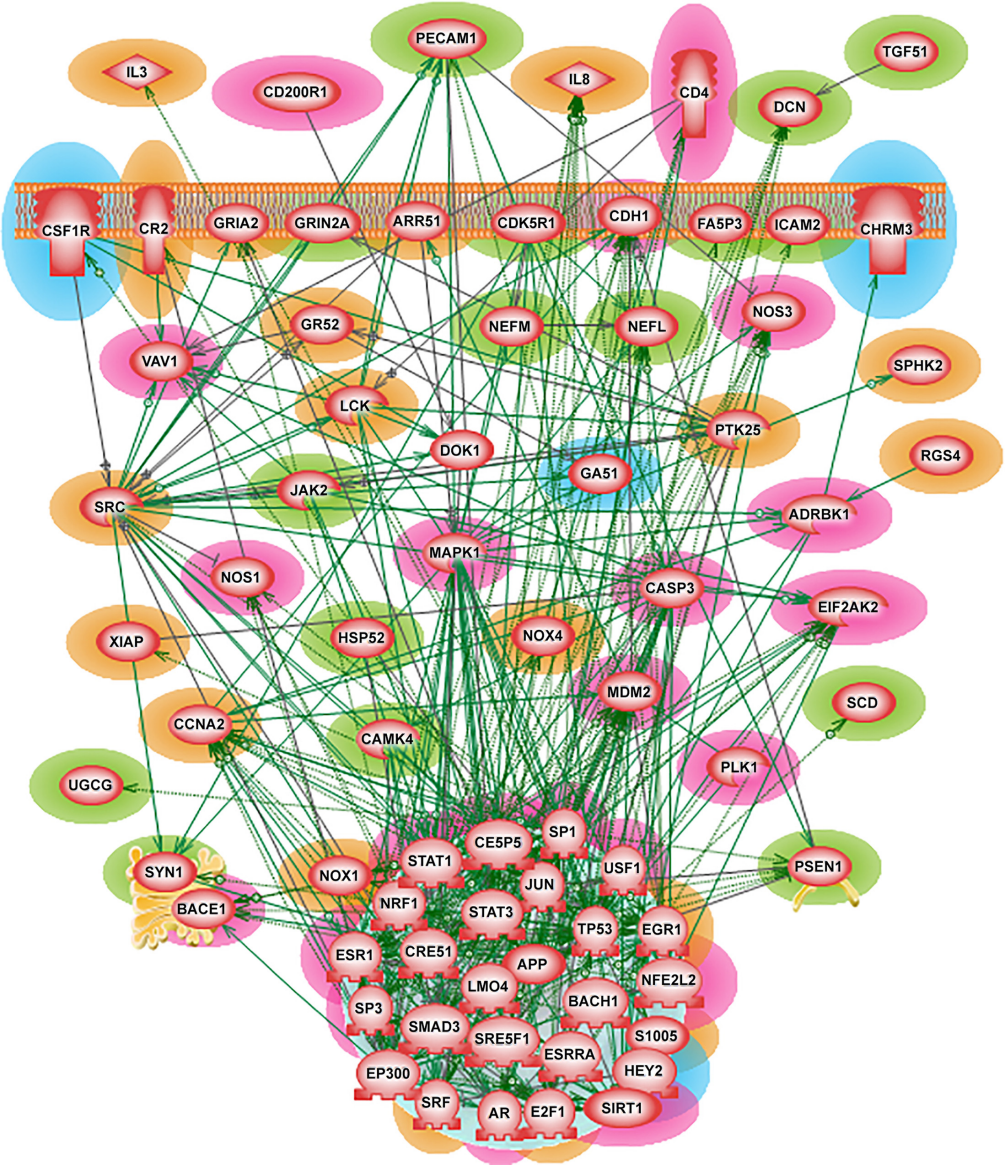
Alzheimer’s disease *compact* shortest path network. The genes/proteins implicated in AD pathology are highlighted in green and red. The genes/proteins of potential interest are highlighted in blue and orange. (see Table 1 for gene highlighting details).

Compared to *direct interaction* network, *shortest-path* network offered a better means to evaluate the complexity and the interconnectedness of the significantly differentially expressed genes in the Alzheimer’s disease. However, the SPNW was still complex to derive any valuable information regarding critical players and molecular mechanisms of AD. We decided to further reduce the network size and construct a *compact* version of the SPNW network that would reveal the key contributors of Alzheimer’s disease. Such a *compact* SPNW was built using the genes summarized in Table 1 along with few additional connecting genes without which some of the genes of interest would remain unconnected. The resulted *compact* SPNW had an average node degree of 8.81 and genes like CREB1, MAPK1, JUN, SP1 and TP53 were highly connected nodes (hubs). In biomolecular networks, hub genes tend to be part of critical functions or pathways (for example CREB1, MAPK1 and TP53). In this network, we found 24 such genes with node degree > 10. Many of these hub genes were also the nodes with high closeness (better accessibility) and betweenness (traffic-influential) centrality scores in this compact SPNW. Fig 3 illustrates the interactions between the known and the genes of interest in the AD *compact* SPNW.

#### Guilt-by-association analysis

The guilt-by-association analysis, based on the immediate proximity to some of the already known AD genes revealed some genes (listed in Table 2) that could be of interest in Alzheimer’s disease paradigm. Further, we explored earlier bimolecular research works on these genes to understand their innate molecular functions and mechanisms, if any.

**Table 2:** Genes of interest for Alzheimer’s disease identified by “guilt-by-association” with the known AD-related genes.

Genes of Interest	Interacts with no. of known AD genes
SRC	**13**
MDM2	**11**
NRF1	5
ESRRA, PTK2B, SRF	4
CR2, NOX4	2
CHRM3, CSF1R, HEY2, IL3	1

SRC (v-src sarcoma (Schmidt-Ruppin A-2) viral oncogene homolog (avian)) a tyrosine kinase is a very generic gene/protein, which controls a wide variety of processes, pathways, and actions, and is responsible for key events in the body, we found it to be the first-level neighbor to 13 already known Alzheimer’s disease genes (ADRBK1, AR, CASP3, CDH1, JAK2, JUN, NFE2L2, NOS1, PECAM1, SP1, STAT1, STAT3 and VAV1). This association with many known AD genes makes SRC a strong candidate for a role in Alzheimer’s pathology. Moreover, it has been demonstrated that accumulation of amyloid-β stimulates microglial (macrophages of brain) activation via SRC tyrosine kinase pathway. Murine AD model study has shown that a cancer drug named Dasatinib could inhibit SRC tyrosine kinase activity of microgliosis in the AD diseased brains. Decreased microglial activity was correlated with improved cognitive performance in mouse [56]. Cell culture study also suggests that SRC tyrosine kinase inhibition shows promising route in mitigating the effects of amyloid-β accumulation in brains which could be tested in human Alzheimer’s disease brains [57].

Based on its high proximity to AD related genes, another important player in AD is MDM2 which is directly connected to eleven already known Alzheimer’s disease genes. MDM2 (p53 E3 ubiquitin protein ligase homolog (mouse)) was shown in 2010 in possible relation to AD in the pathway map of Proctor and Gray [58]. It has E3 ubiquitin ligase activity, which targets tumor suppressor protein p53 for proteasomal degradation. It was shown that amyloid-β signaling pathway disrupts biochemical pathways involving lipid metabolism, ultimately affecting tau phosphorylation which contributes to AD [59]. Perturbation of lipid metabolism, especially of cholesterol homeostasis, has been illustrated in an another severe progressive neurodegeneration called Niemann-Pick Type C (NPC) disease [60]. In a mouse NPC model, it has been demonstrated that cholesterol perturbation-induced axonal growth cone collapse and decrease in phosphorylated p53 were reduced by inhibition of MAPK and MDM2 E3 ligase.

Another proposed candidate gene is NRF1which neighbors with five (CREB1, ESR1, NFE2L2, NOS1 and TP53) genes involved in Alzheimer’s disease mechanism. One of the distinctive characteristic features of several neurodegenerative diseases is the aberrant accumulation of protein (like α-synuclein, amyloid-β) aggregations. This protein buildup leads to mitochondrial dysfunction which eventually increases the ROS production in the brain [49,61]. Both NRF1 and NRF2 are transcription factors that play a pivotal role in the protection against the toxic effects of reactive oxygen species (ROS). Studies have suggested that NRF1 and NRF2 promote mitochondrial biogenesis as well as play crucial role in neuronal survival after acute brain injury [62,63]. Our finding for NRF1 as a potential important factor in AD, reported in May 2013 during the defense of the PhD Thesis of Dr. Chandrasekaran, was confirmed five months later in the independent study of Satoh et al. [64] based on analysis of ChIP-Seq set of genes. Summarizing the analysis done in the foregoing it is fair to conclude that, due to their neighborhood connectivity, as well as by their known physiological roles, the genes listed in Table 2 appear as credible genes of potential importance in Alzheimer’s disease, needing experimental validation.

#### Molecular functions and network attributes of some of the critical connecting genes

Other than the potential candidate genes, the *compact* SPNW also included many significant connecting proteins like APP, BACE1, CCNA2, CREB1, E2F1, EGR1 and MDM2 which were previously implicated to play critical roles in many neurodegeneration disease pathogenesis including Alzheimer’s. Abnormal proliferation of APP is the major contributor of the neurodegeneration process in AD. Pathogenic alterations of BACE1, CCNA2, CREB1, E2F1 and EGR1 are evident in the disease progression. BACE1 initiates the amyloid-β formation [65]. In general, cyclins (CCNA2) were shown to increase the severity of Alzheimer-related pathology and other types of dementia [66]. In addition, cyclins are regulators of CDK kinases. It has been indicated that APP and tau, the major two proteins implicated in AD, are the physiological substrates for cyclin-dependent kinase especially by a neuron-specific cyclin kinase called CDK5 [67]. Similarly, E2F1 were found co-localized with amyloid-β plaques. It may ultimately contribute to neuronal cell death in AD [68]. CREB1 along with CREM were suggested to play major therapeutic role in neurodegeneration [69].

Apart from having pivotal role in neurodegeneration mechanism, the above mentioned connecting genes interact with many previously known-AD genes. Except BACE1, all other genes are among the top 30 nodes with highest connectivity (degree > 8) in the *compact* shortest-path network. CREB1 turns out to be one of the critical nodes in the network. It has many advantages being one of the top five highly connected nodes, having better visibility as measured the closeness centrality as well as by its capacity to control the flow of traffic within the network as assessed by the betweenness centrality.

#### DAVID enrichment analysis

Additionally, we also carried out gene set enrichment analysis using DAVID software tool to identify different biological processes and/or pathways affected in Alzheimer’s disease. Table 3 lists some of the Gene Ontology categories/subcategories related to nervous system and functions that were statistically significantly enriched in AD (with Benjamini-Hochberg multiple correction).

**Table 3:** Gene set DAVID enrichment analysis of Alzheimer’s disease *compact* shortest-path network.

Term	Gene Count	Fold Enrichment	Benjamini
GO:0030424~axon	13	15.60	4.84E-09
GO:0043005~neuron projection	15	8.37	1.58E-07
GO:0007568~aging	8	13.48	8.02E-05
GO:0043523~regulation of neuron apoptosis	7	14.41	2.76E-04
GO:0030425~dendrite	8	9.36	3.87E-04
GO:0030182~neuron differentiation	12	5.08	5.43E-04
GO:0007169~transmembrane receptor protein tyrosine kinase signaling pathway	9	7.45	6.43E-04
GO:0006979~response to oxidative stress	8	9.04	6.66E-04
GO:0042325~regulation of phosphorylation	12	4.77	8.15E-04
GO:0001944~vasculature development	9	6.64	0.001
GO:0045202~synapse	10	5.37	0.001
GO:0048666~neuron development	10	5.47	0.002
GO:0031594~neuromuscular junction	4	34.69	0.002
GO:0007611~learning or memory	6	10.02	0.005
GO:0031175~neuron projection development	8	5.79	0.006
GO:0008637~apoptotic mitochondrial changes	4	23.91	0.008
GO:0000302~response to reactive oxygen species	5	12.35	0.009
GO:0007005~mitochondrion organization	6	8.06	0.010
GO:0035235~ionotropic glutamate receptor signaling pathway	3	61.77	0.011
GO:0007268~synaptic transmission	8	4.97	0.011
GO:0007259~JAK-STAT cascade	4	19.01	0.013
GO:0019226~transmission of nerve impulse	8	4.24	0.024
GO:0007409~axonogenesis	6	5.76	0.032
GO:0008088~axon cargo transport	3	30.89	0.035
GO:0050877~neurological system process	15	2.30	0.036
GO:0051402~neuron apoptosis	3	29.26	0.038
GO:0048667~cell morphogenesis involved in neuron differentiation	6	5.32	0.042
GO:0048812~neuron projection morphogenesis	6	5.22	0.045

DAVID analysis revealed some of the biological processes like oxidative stress, neuron apoptosis, tyrosine kinase signaling pathway, regulation of phosphorylation and aging affected in Alzheimer’s disease, which were also discussed above as part of AD mechanisms. It is valuable to note that the ALS pathway, characterizing another debilitating neurodegenerative condition called Amyotrophic lateral sclerosis, was also enriched among Alzheimer’s disease genes. Enrichment of ALS disease pathway along with Huntington’s disease pathway was reported by Ingenuity’s IPA analysis. One more promising step towards our study goal of identifying and reporting common genes and pathways in major neurodegenerative disorders was thus achieved.

### *Integrated* Alzheimer’s disease mechanism

The results of DAVID analysis were further evaluated in an attempt to elucidate details of the underlying molecular mechanism of Alzheimer’s disease. DAVID analysis revealed 22 KEGG pathways that were significantly (p-value <0.05 with Benjamini-Hochberg multiple correction) affected in AD. These pathways were found to take part in signal transduction, immune system, cell communications, nervous system and in general neurodegenerative diseases categories. Twelve out of 22 enriched KEGG pathways (listed in Table 4) were selected and the genes from these pathways were then consolidated into a list of 37 common genes. The pathways selection was helped by previous Alzheimer’s disease research literature reviews [70–81].

**Table 4:** Enriched KEGG pathways in Alzheimer’s disease resulted from DAVID analysis.

Term	Gene Count	Fold Enrichment	Benjamini	Genes
hsa05014:Amyotrophic lateral sclerosis (ALS)	7	12.21	6.67E-04	CASP3, NOS1, GRIA2, GRIN2A, TP53, NEFL, NEFM
hsa04062:Chemokine signaling pathway	10	4.94	0.002	MAPK1, IL8, GRB2, ARRB1, PTK2B, JAK2, ADRBK1, STAT1, VAV1, STAT3
hsa04722:Neurotrophin signaling pathway	7	5.22	0.014	MAPK1, PSEN1, CAMK4, GRB2, JUN, GAB1, TP53
hsa05010:Alzheimer's disease	8	4.54	0.015	MAPK1, APP, CDK5R1, CASP3, NOS1, PSEN1, BACE1, GRIN2A
hsa04650:Natural killer cell mediated cytotoxicity	7	4.87	0.017	MAPK1, CASP3, GRB2, PTK2B, ICAM2, LCK, VAV1
hsa04720:Long-term potentiation	5	6.80	0.029	MAPK1, EP300, CAMK4, GRIA2, GRIN2A
hsa04660:T cell receptor signaling pathway	6	5.14	0.030	MAPK1, GRB2, JUN, LCK, CD4, VAV1
hsa04662:B cell receptor signaling pathway	5	6.16	0.034	MAPK1, CR2, GRB2, JUN, VAV1
hsa04520:Adherens junction	5	6.00	0.036	MAPK1, EP300, SMAD3, CDH1, SRC
hsa04020:Calcium signaling pathway	7	3.68	0.040	NOS1, CAMK4, SPHK2, CHRM3, PTK2B, GRIN2A, NOS3
hsa04012:ErbB signaling pathway	5	5.31	0.049	MAPK1, GRB2, JUN, GAB1, SRC
hsa04350:TGF-beta signaling pathway	5	5.31	0.049	MAPK1, EP300, SP1, SMAD3, DCN

#### KEGG analysis

**Fig 4:**
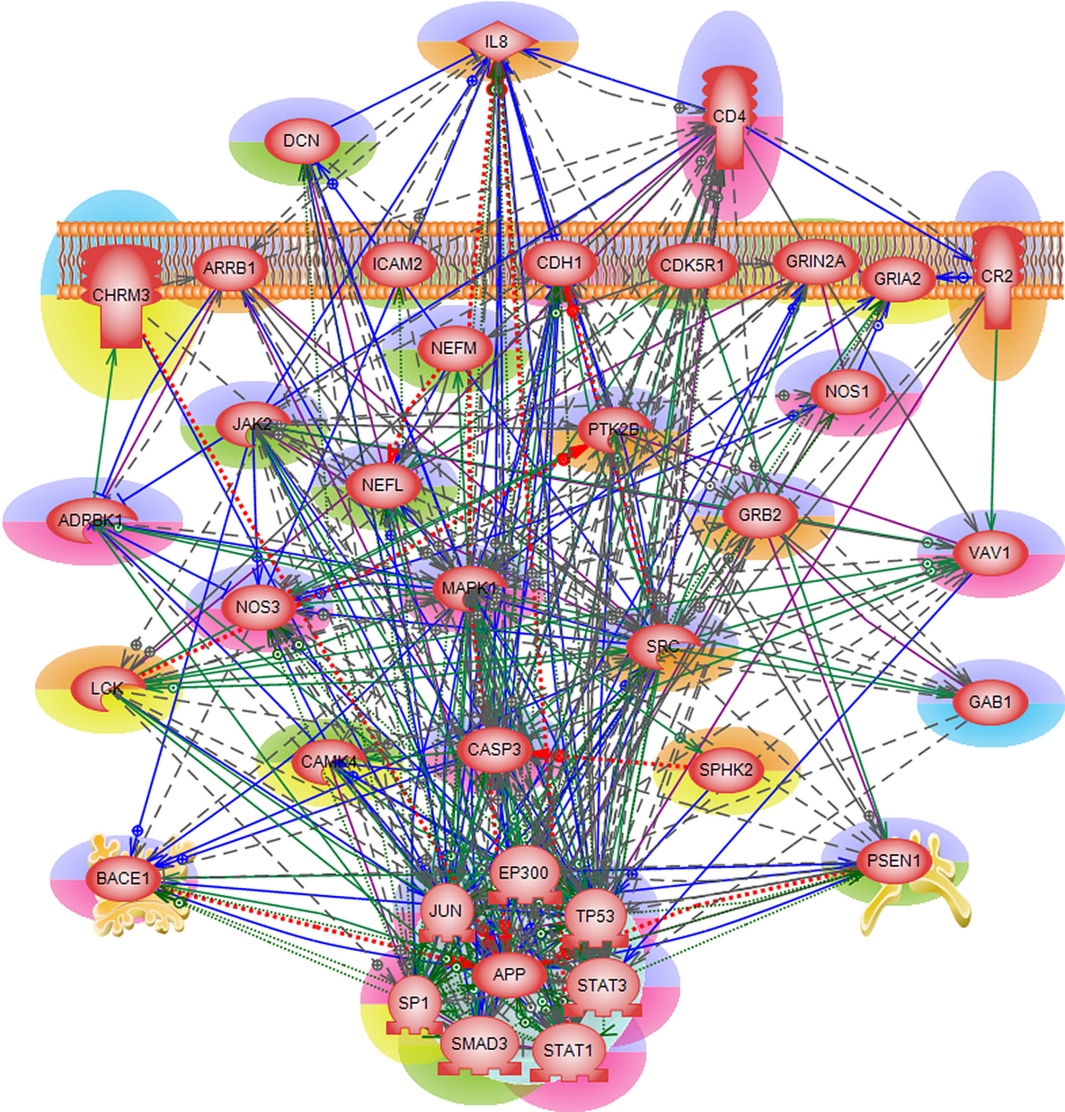
*Integrated* Alzheimer’s disease mechanism network. The 37 genes/proteins are found in common in all 12 enriched KEGG pathways. Genes/proteins implicated in AD pathology are highlighted in green/red and the genes/proteins of potential interest are highlighted in blue/orange (See Table 1. for details). Genes/proteins causing neuronal loss are highlighted in purple and those that help in neuronal survival are in yellow.

An *integrated* Alzheimer’s disease mechanism network was constructed (see Fig 4) to analyze the interactions among these 37 common genes. We found a number of routes of disease initiation via three extra-cellular proteins namely, CD4, DCN and IL8. Here, we summarize some of the biological functions of the three proteins and their role in Alzheimer’s disease pathogenesis. Increased accumulation of amyloid-β plaques in AD brain has been shown to attract a surge of inflammatory mediators including chemokines, cytokines and interleukins leading to chronic inflammation that triggers neuronal death [82]. IL8 (interleukin 8) is a member of the CXC chemokine family and one of the major mediators of the inflammatory response. Studies have shown overproduction of IL8 along with other interleukins found in specific regions affected in Alzheimer’s disease. Under normal conditions, IL8 promotes cell-cell communications in central nervous system, but increased amyloid-β production leads to chronic stimulation of IL8 response which is suspected as cause for abnormal physiology in AD brains [83]. Similarly, overproduction of CD4 has been associated with indirect neuronal damage in infectious and immune- mediated diseases of the central nervous system. Persistent amyloid-β stimulus in AD brains has been implicated in chronic CD4 activation which results in premature immunosenescence (gradual deterioration of the immune system brought on by natural age advancement) manifestation [84]. DCN (decorin) is a small cellular or pericellular matrix proteoglycan. Earlier immunohistological study of AD brain tissues has found that decorin was primarily localized to the periphery of the amyloid plaque and to the edges of amyloid fibril bundles within the plaque periphery. It has been suggested that decorin molecules may contribute to regulating the size of amyloid accumulation and/or in maintaining their spherical shape [85]. In order to achieve continued homeostasis, a delicate balance has to be maintained between APP gene expression and various inflammatory mediators.

Thus, the three extra-cellular molecules of the integrated AD mechanism network have already been implicated in Alzheimer’s disease pathogenesis in one way or another. The *integrated* network uncovered *direct* interactions between these three extra-cellular molecules and amyloid- β (A4) precursor protein, APP inside the nucleus. In addition, the network revealed interdependent interactions between APP, BACE1 and PSEN1 genes/proteins. Deregulation of APP, BACE1 (beta-site APP-cleaving enzyme 1) and PSEN1 are well-known causes of Alzheimer’s disease where APP and PSEN1 mutations are associated with familial form of the disease and BACE1 gene codes for one of the proteases that cleaves APP whose accumulation is the hallmark of Alzheimer’s disease. Apart from *direct* interactions with APP, BACE1 and PSEN1, the three extra-cellular molecules also trigger the downstream targets of MAP kinases, and caspases directing cell death.

#### Classification of disease causing and therapeutic mediators

**Fig 5:**
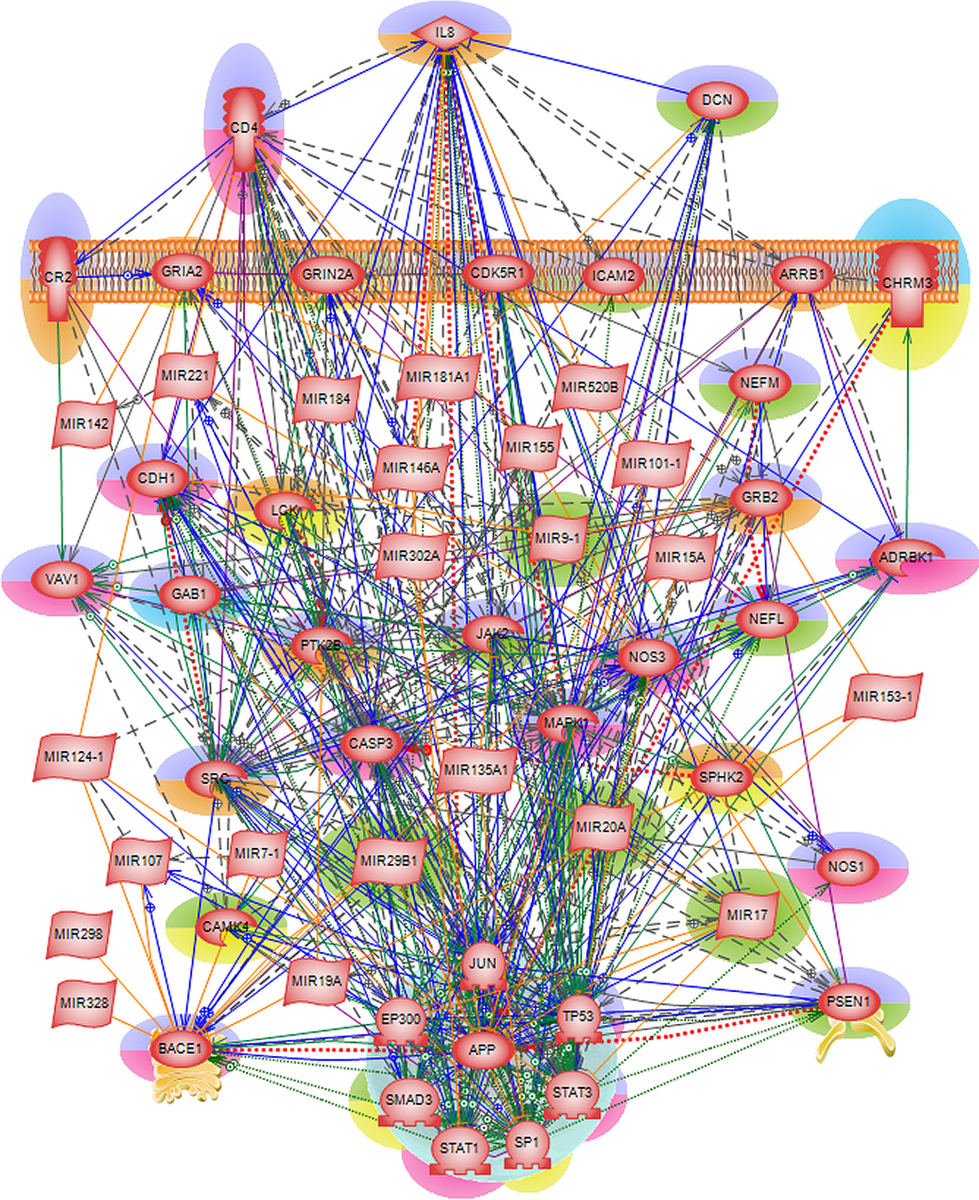
MicroRNA incorporated *integrated* Alzheimer’s disease mechanism. The 37 genes/proteins found in common in all 12 enriched KEGG pathways are regulated by 22 microRNAs. Genes/proteins implicated in AD pathology are highlighted in green/red and the genes/proteins of potential interest are highlighted in blue/orange (See Table 1) for details. Genes/proteins causing neuronal loss are highlighted in purple and those that help in neuronal survival are in yellow.

Next, each of the 37 common genes of the enriched KEGG pathways was subjected to Google and NCBI’s PubMed search for their biological/molecular functions, as well as for their potential role in AD pathogenesis. Based on this search, we categorized these 37 genes as either disease causing (leading to neuronal death) or possible therapeutics (helping with reducing the harmful effects and/or preventing neuronal loss) for Alzheimer’s disease. This categorization is depicted in Fig 5 where the genes are highlighted either in purple or yellow, respectively.

As mentioned earlier, by modulating SRC tyrosine kinase gene expression we could employ some inhibition of amyloid-β accumulation in AD brains. But in general, the *integrated* disease mechanism network resulted into a well-connected destructive pathway with almost 80% of the 37 common genes found to play harmful roles in AD pathogenesis. We could try exerting gene/protein expression regulation through some of the transcription factor in the integrated network. However, the transcription factors found in this network were very generic and they are required for normal functioning of the cell. Thus, it was difficult to find/propose a node in the *integrated* Alzheimer’s disease mechanism network through which we could modulate the expression of disease causing/aggravating genes. It was mentioned before that the amyloid-β production has to be delicately balanced in order to maintain homeostasis within the cell. The *integrated* network revealed none to very little room to modulate APP gene/protein expression.

#### Proposed treatment options

We then sought after expanding the underlying AD mechanisms by superimposing the level of fine gene expression regulation by microRNAs, the short non-coding RNAs that regulate gene expression post-transcriptionally. Dysregulation of miR-125b, miR-9, miR-210 and others have been consistently reported in several miRNA expression profiling of AD-affected brain studies [86–88]. Even though many of microRNA’s biological functions are still unclear, they offer many attractive features from a drug development standpoint such as small size, specific regulation, conserved nucleotide sequence etc., which makes them potential therapeutic entities. Currently, microRNAs as medical treatment means in various disease conditions are pursued either through the use of synthetic active microRNAs called mimics or through the use of anti-microRNAs. For example, miR-208 is used in cardiac diseases, miR-155 in chronic inflammatory diseases and miR-122 in Hepatitis C Virus [89]. In AD, overexpression of miR-195 can potentially be used as a therapeutic strategy [90]. A recent AD mouse model study has shown that noninvasive intranasal delivery of AM206, a neutralizing inhibitor of miR-206 has increased brain-derived neurotrophic factor (BDNF) level in brain [91]. In general, BDNF gene regulates synaptic plasticity and memory. Many microRNA clinical trials are also ongoing.

Pathway Studio’s ResNet 9.0 database was used to identify the microRNAs that target APP, BACE1, PSEN1, as well as the three extra-cellular molecules CD4, DCN and IL8. Twenty two such microRNAs were found including five of the already AD-known microRNAs namely, miR-153, miR-17, miR-20a, miR-29b-1 and miR-9. For example, BACE1 (beta-site APP-cleaving enzyme 1) is the target gene for miR-29a/b-1. BACE1 enzyme is essential for the generation of amyloid-β, the hallmark neuropathological lesions in Alzheimer’s disease brain. Earlier study shows that loss of miR-103, miR-29a/b-1 and miR-9 can contribute to increased BACE1 and Aβ levels in sporadic AD [92]. In addition to APP and BACE1 gene expression regulation, miR-9 was suspected to regulate PSEN1, a familial early-onset AD gene. Loss of PSEN1 impacts memory, synaptic plasticity and induces neurodegenerative changes (see above for more details). In a PSEN1 null mice model study, miR-9 down-regulation was correlated to severe brain defects [93]. Using transgenic mouse model of AD, it was found that miR-153 downregulated the expression of APP and APLP2 (a homologue of APP) proteins [94]. Another study strongly suggests that miR-17-5p along with miR-20a and miR-106b may contribute, at least in part, to the developmental regulation of APP expression in brain and in differentiating neurons [95].

With possible therapeutic role of microRNAs in mind, we incorporated the 22 microRNAs into our *integrated* Alzheimer’s disease mechanism network (see Fig 5). Following this we examined the microRNAs and their targets. BACE1 had the highest microRNA regulation potential being targeted by nine miRNAs, next were APP and CD4, each of which was targeted by five microRNAs, then IL8 with three microRNA regulations, and one each for PSEN1 and CD4. In addition, miR-320a was found to target both APP and IL8, similarly miR-181a1 targeted both CD4 and DCN. In conclusion, by incorporating microRNAs into the *integrated* network, we were able to suggest some critical nodes to regulate Alzheimer’s disease causing and/or aggravating genes. Using network techniques we could offer both narrow and specific range of entities through which the network could be modulated. Thus, we propose 17 microRNAs (miR-101-1, miR-107, miR-124-1, miR-135a1, miR-142, miR-146a, miR-155, miR-15a, miR-181a1, miR-184, miR-19a, miR-221, miR-298, miR-302a, miR-328, miR-520B and miR-7-1) in addition to the known AD-related five as potential therapeutic targets in Alzheimer’s disease. Most of these novel microRNAs are not yet experimentally verified but one may expect that after such verification a sizable part of them will be found of therapeutic interest.

### Alzheimer’s disease *microRNA* regulatory network

**Fig 6:**
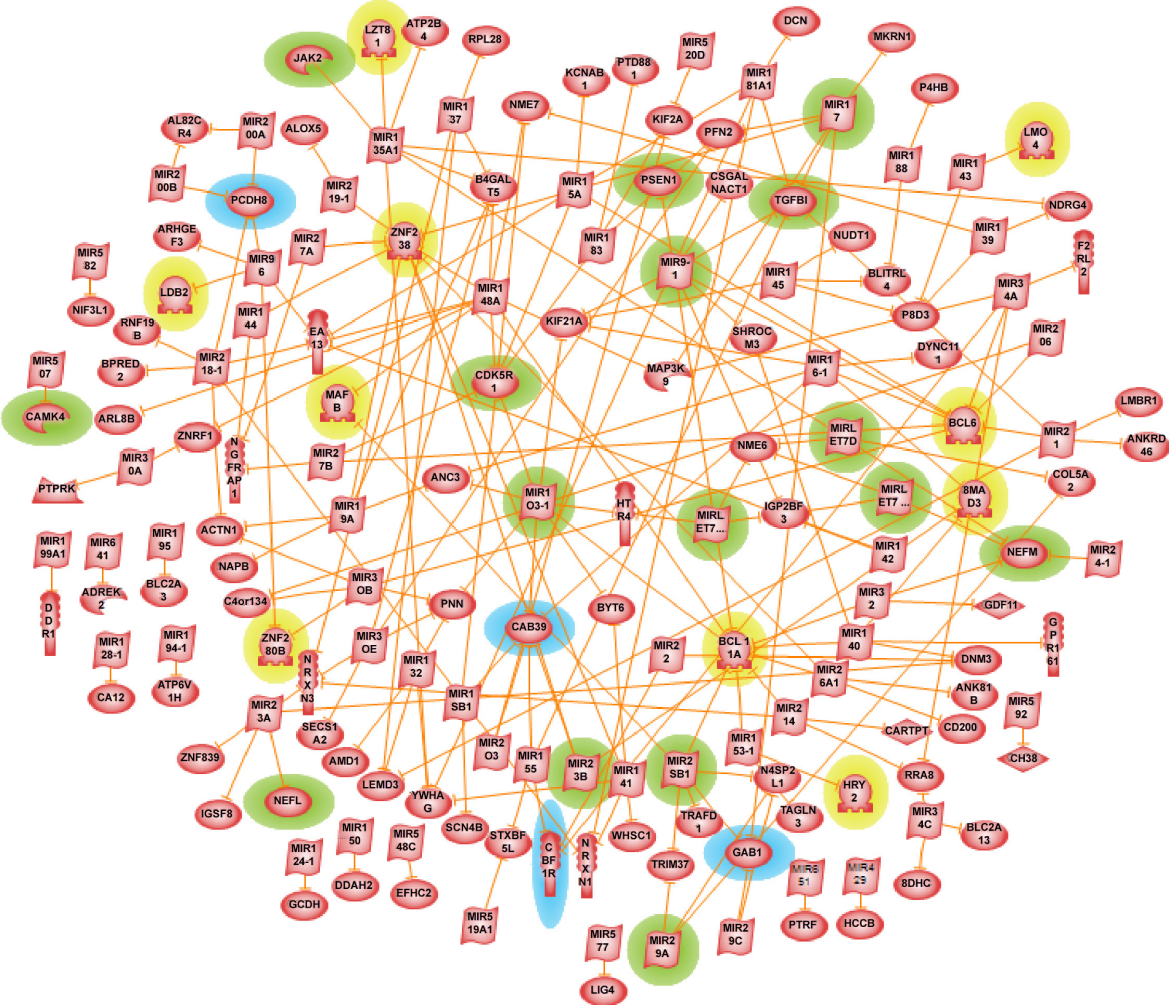
Alzheimer’s disease *miRNA* regulatory network. The genes/proteins and miRNAs implicated in AD pathology are highlighted in green and the genes/proteins of potential interest are highlighted in blue. Genes/proteins that code for transcription factors (TFs) are highlighted in yellow.

Besides finding the microRNAs that could regulate the critical nodes such as APP, BACE1, CD4, DCN, IL8 and PSEN1, we searched to uncover additional regulatory mechanisms of Alzheimer’s disease genes. For this we used all the 214 significantly differentially expressed AD genes and constructed a shortest-path network with only microRNA interactions found in Pathway Studio’s ResNet 9.0 database. This SPNW revealed that 67 miRNAs regulate many of the 214 AD SDEGs. Utilizing these 67 miRNAs and 214 SDEGs, we then constructed a *direct* miRNA-mRNA targets interaction network shown in Fig 6.

In addition to miRNAs regulation, the network also incorporated regulation by ten transcription factors like BCL6, LMO4, HEY2 and SMAD3 which were not among the very generic transcription factors (TFs) reported earlier. Thus, like in other complex diseases, cooperative two-level gene expression regulatory mechanisms occur in Alzheimer’s disease pathogenesis via miRNAs and transcriptional factors. On further examination of the *microRNA* regulatory network (Fig 6), miR-148a was the node with the highest degree (hub), closeness and betweenness centrality scores. It regulates gene expression of ten genes including CDK5R1 whose expression is needed for proper CDK5 activity, as a part of the AD molecular mechanism discussed above. MicroRNA quantitative biochemical study has reported altered expression of miR-148a along with other microRNAs in hippocampus and medial frontal gyrus regions of human AD brain samples [96]. In addition, our network analysis revealed that expression of CDK5R1 gene could also be regulated by five microRNAs (miR-103, miR-148a, miR-15a, miR-183, and miR-27b), of which regulation via miR-103 along with miR-107 has already been experimentally established [97].

Additional microRNA regulation of some of the genes of interest in Alzheimer’s disease was uncovered in the *microRNA* regulatory network. It includes modulation of CAB39 gene expression by six microRNAs (miR-103-1, miR-155, miR-16-1, miR-19b1, miR-203 and miR-23b), PCDH8 by four microRNAs (miR-200a/b, miR-218-1 and miR-96), CSF1R by three microRNAs (miR-155, miR-22 and miR-34a), GAB1 by three microRNAs (miR-17, and miR-29b1/c), CAMK4 by miR-507 and HEY2 by miR-153-1, which would require experimental validation. Finally, we found that miR-148a, miR-155 and miR-153 seem to target many of the critical genes, as well as genes of interest, in Alzheimer’s disease.

## Discussion

Online Mendelian Inheritance in Man (OMIM) database search for Alzheimer’s disease revealed many genes such as A2M, ACE, APOE, APP, NOS3, PSEN1, PSEN2 and SORL1 implicated in the disease mechanism. However, none of these genes except PSEN1 were included in our “seed genes” since these genes were not strongly differentially expressed, and also their log-fold change was minimal (ranged from -0.3 to +0.1). We consider the lowered gene expression intensity in the post-mortem brain samples compared to a functioning brain as attributing factor to this gene set low variability. But it is noteworthy to note that APP and NOS3 along with other well-known AD genes like AR, BACE1, STAT1 etc., although not significantly changed were later included as connecting genes/proteins in our *shortest-path* network. This turned those genes into critical nodes in the closest network environment of the known significantly changed Alzheimer’s genes. These findings reflect upon our ideas that it is beneficial to utilize network techniques to study complex systems like neurodegenerative diseases where even in the absence of an appropriate study medium (a fully functional brain) we could *recreate* some of missing links/nodes that are valuable to understand the underlying system mechanism.

It should be mentioned that our detailed network analysis was unable to confirm the AD role of APOJ, TIMP3 and IRAK1 genes reported by Dunckley et al. 2006 [31]. These genes were not statistically significant after the normalization we performed on their dataset, and they did not emerge in any way in the networks we constructed on the basis of well-established AD genes. We may also note that two genes having a significant role in oxidative stress could not be included in our network analysis. These are DRP1, which is not part of the microarray probeset, and VDAC1 which is neither significantly modulated in AD, nor could it satisfy the other criteria used for selection of the seed genes in our analysis. In contrast, the identification of PSEN1, a well-known familial Alzheimer’s disease gene as an important node in both *direct* interaction and *shortest-path* network is critical in number of ways. First, PSEN1 was found to be significantly differentially expressed gene (p-value < 0.01) in the microarray datasets. Secondly, it directly interacts with many already known AD genes such as APP, CDH1, CDK5R1, CREB1, JUN, SP1 and STAT3. It is also among the prioritized top 33 nodes with degree ≥ 8, and top 26 nodes with high closeness centrality index (high accessibility to all other nodes) in the *compact* shortest-path network, emerging thus with a major role in the molecular mechanism of Alzheimer disease.

Our network analysis provided sufficient arguments in favor of GAB1 as novel candidate genes for Alzheimer’s disease based on its direct network interactions with already known AD genes. With somewhat less confidence we propose also CSF1R gene, which despite being a second-level neighbor to AD related genes, has demonstrated important role in neuronal survival after injury and degeneration. Both genes are significantly differentially expressed in the post-mortem AD samples we used and they could have critical role as neuroprotective agents. From shortest-path network assessment, being first-level interacting partners with three or more already known AD-related genes we conclude that *connecting* genes/proteins added by the Pathway Studio software such as ESRRA, NRF1, PTK2B, SRC and SRF could also be of potential interest in Alzheimer’s disease domain. Among the above listed genes, SRC was *directly* connected with at least ten or more previously known AD genes. Apart from *directly* interacting with a large number of known AD-related genes/proteins as shown in the guilt-by-association list above, SRC is among the top 25 hub nodes (with degree > 10), among the top 25 nodes with higher accessibility to all other nodes (as measured by closeness centrality network descriptor) and among the top 25 traffic influential nodes in the network (as measured by the betweenness centrality index). All this may be considered as evidence for a potential important systems biology role of SRC gene in the integral mechanism of Alzheimer’s disease. Additionally, one may consider including to the list of Alzheimer’s genes five more from the connecting genes in the *shortest-path* network (CHRM3, CR2, HEY2, IL3 and NOX4) via their guilt-by-associations to one or two already known AD genes. The ARRB1 and SPHK2 *connecting* genes, which are second-level interacting partners with previously implicated AD genes, may also be viewed of interest via their role as modulators in amyloid-β production [98,99].

In addition to identifying novel individual genes, proceeding from our *integrated* Alzheimer’s disease mechanism network, we propose three disease initiating routes via extra-cellular ligands like CD4, DCN and IL8. These genes directly interact with many known AD genes including APP, BACE1 and PSEN1 and influence AD pathogenesis mechanism. Since this network was well-connected with almost 80% of genes promoting neurodegeneration process, we were unable to propose any therapeutic targets or mechanisms using this network. However, by incorporating *microRNA* regulations to this network, we identified 17 microRNAs (miR-101-1, miR-107, miR-124-1, miR-135a1, miR-142, miR-146a, miR-155, miR-15a, miR-181a1, miR-184, miR-19a, miR-221, miR-298, miR-302a, miR-328, miR-520B and miR-7-1) in addition to the known five AD-related miRNAs as potential therapeutic targets in Alzheimer’s disease. More generally, we propose a scheme of complex multi-level regulations taking place between the critical players of AD, such as APP, BACE1, and PSEN1, and other disease causing/alleviating entities. In order to maintain continued homeostasis, a delicate balance should be sustained between the genes, and with its surgically accurate mode of action the microRNA regulation could play an important role in this scenario. Perhaps, a well-designed cellular automata simulation would help to shed some light on this delicate but life-determining balance. The genes discussed in the summary were also shown by gene ontology analysis to be key players in numerous specific biological mechanisms like neuroprotection against amyloid-β accumulation, formation of neurofibrillary tangles, synapses, cognitive deficits, mental retardation, memory loss and attenuating APP accumulation, many of which are known to be modulated in the underlying Alzheimer’s pathophysiology and potential compensatory responses.

Summarizing our study it is noteworthy mentioning that the successful identification of novel important disease-related genes requires a combination of gene expression modulation data with the knowledge accumulated in databases for biomolecular interactions. This systems biology approach makes possible the construction of diverse types of informative networks whose topological analysis provides reliable basis for prioritizing of network nodes based on their connectivity and centrality. In parallel, accounting for the neighborhood relations to already experimentally confirmed disease-related genes increases the chance for successful predictions, based on the guilt-by-association rule. In the search for molecular mechanisms of Alzheimer’s disease a different route seems to be fruitful. It proceeds with building an integrated mechanistic network by overlapping the known disease-related KEGG molecular pathways, adding more interactions between the network nodes, again provided by databases of such interactions. Identifying novel AD-related microRNAs adds more options in the search for beneficial regulatory molecular mechanisms. We intend applying such strategy in a future publication devoted to the search of common unifying mechanisms of neurodegenerative diseases.

## Supporting Information

S1 Fig Biological processes and genes implicated in the Alzheimer’s disease. Courtesy: Alzheimer’s disease pathway from KEGG database, retrieved on Apr 3, 2013. Publicly available at http://www.genome.jp/kegg-bin/show_pathway?hsa05010.
(TIF)

S1 Table List of 214 significantly differentially expressed genes (SDEGs). (XLSX)
