# Correction: Network Topology Analysis of Post-Mortem Brain Microarrays Identifies More Alzheimer's Related Genes and MicroRNAs and Points to Novel Routes for Fighting with the Disease

**DOI:** 10.1371/journal.pone.0151122

**Published:** 2016-03-09

**Authors:** The PLOS ONE Staff

Fig 6 appears incorrectly in the published article. Please see the correct Fig 6 and its legend here. The publisher apologizes for the error.

**Fig 6:**
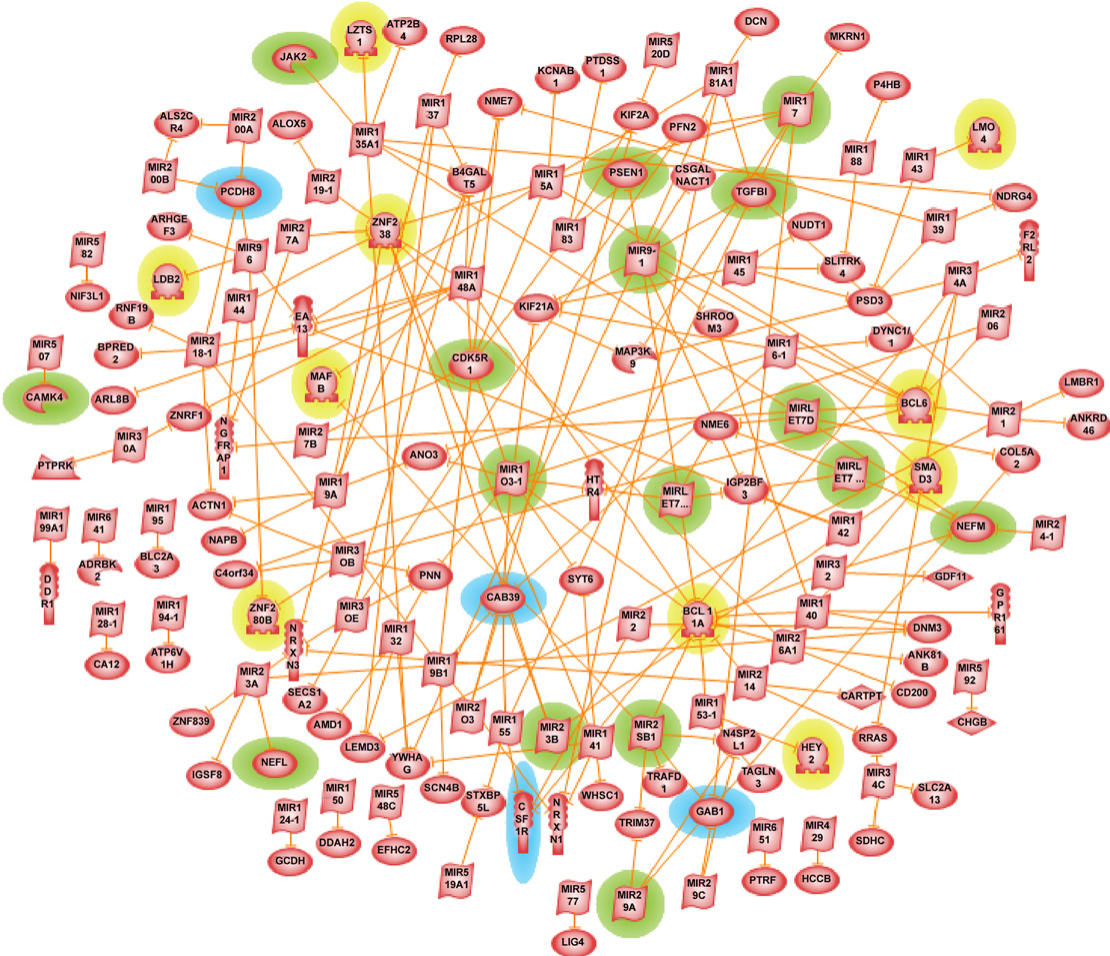
Alzheimer’s disease miRNA regulatory network. The genes/proteins and miRNAs implicated in AD pathology are highlighted in green and the genes/proteins of potential interest are highlighted in blue. Genes/proteins that code for transcription factors (TFs) are highlighted in yellow.
