# Supplementary figures and images for: Network Topology Analysis of Post-Mortem Brain Microarrays Identifies More Alzheimer’s Related Genes and MicroRNAs and Points to Novel Routes for Fighting with the Disease
Source: PLoS One. 2016 Jan 19;11(1):e0144052. doi: 10.1371/journal.pone.0144052 (PMC4718516; doi:10.1371/journal.pone.0144052)

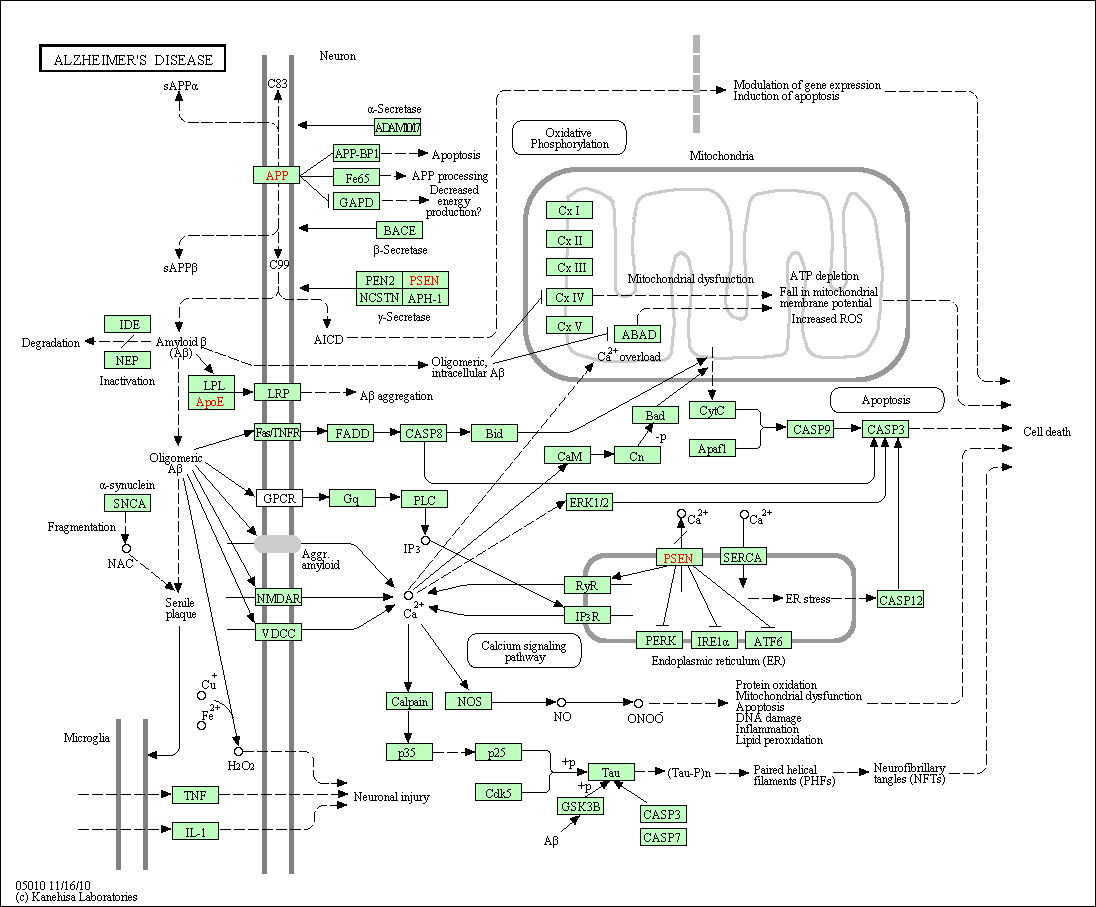

Supplement: S1 Fig — Courtesy: Alzheimer’s disease pathway from KEGG database, retrieved on Apr 3, 2013. Publicly available at http://www.genome.jp/kegg-bin/show_pathway?hsa05010. (TIF) [file pone.0144052.s001.tif]
